# Biocontrol Potential of Purified Elicitor Protein PeBL1 Extracted from *Brevibacillus laterosporus* Strain *A60* and Its Capacity in the Induction of Defense Process against Cucumber Aphid (*Myzus persicae*) in Cucumber (*Cucumis sativus*)

**DOI:** 10.3390/biology9070179

**Published:** 2020-07-21

**Authors:** Khadija Javed, Humayun Javed, Dewen Qiu

**Affiliations:** 1 State Key Laboratory for Biology of Plant Diseases and Insect Pests, Institute of Plant Protection, Chinese Academy of Agricultural Sciences, No. 12 Zhong-Guan-Cun South Street, Beijing 100081, China; khadijajaved829@gmail.com or; 2 Department of Entomology, Pir Mehr Ali Shah Arid Agriculture University, Rawalpindi 46000, Pakistan; hjhumayun@gmail.com

**Keywords:** PeBL1, *Myzus persicae*, aphid resistance, electrical penetration graph, defense pathways, jasmonic acid, salicylic acid, ethylene

## Abstract

The Cucumber aphid (*Myzus persicae*), a destructive cucumber aphid usually managed by chemical pesticides, is responsible for enormous annual agricultural losses. A protein elicitor, PeBL1, was investigated in the present work for its ability to induce a defense response against *M. persicae* in cucumber. The rates of population growth (Intrinsic rate of increase) of *M. persicae* (second and third generations) decreased with PeBL1-treated cucumber seedlings as compared to positive (water) and negative 70.58 μg mL^−1^ controls (50 mM Tris-HCl, pH 8.0). In an assay on host selection, *M. persicae* had a preference for colonizing control plants as compared to the PeBL1-treated cucumber seedlings. The nymphal development time of the aphid was extended with the PeBL1-treated cucumber seedlings. Likewise, fecundity was reduced, with less offspring produced in the PeBL1-treated cucumber seedlings as compared to the positive (water) and negative 70.58 μg mL^−1^ controls (50 mM Tris-HCl, pH 8.0). The cucumber leaves treated with PeBL1 had a hazardous surface environment for *M. persicae*, caused by trichomes and wax formation. Jasmonic acid (JA), salicylic acid (SA), and ethylene (ET) levels were significantly higher, exhibiting significant accumulation in the PeBL1-treated cucumber seedlings. The following results showed that PeBL1 considerably altered the height of the cucumber plant and the surface structure of the leaves to minimize *M. persicae* reproduction, and it prevented colonization. Defensive processes also included the activation of pathways (JA, SA, and ET). This study provides evidence of biocontrol for the use of PeBL1 in cucumber defense against *M. persicae*.

## 1. Introduction

A multifaceted relationship has been established between herbivores and plants during development. Plants damaged by herbivores show the accumulation of toxic or volatile organic compounds with the modification of their physical structures. The structures and compounds affect the growth, colonization, feeding, survival, and oviposition of herbivores, and they attract natural enemies and encourage them to induce defense [1]. In order to deal effectively with this damage, two mainly constitutive defense mechanisms have been developed by plants [2]. Plants are prevented from colonizing by physically impaired barriers, including cuticle trichomes, callose, cell walls, and suberin, while antibiotic allelochemicals affect or induce pest production, fertility, and insect durability [3]. Aphids are phloem-feeding insects that spread plant viruses through the ingestion of plant sap, resulting in severe crop losses [4,5]. In various aphid–plant systems, defense responses caused by aphids have been studied. *Arabidopsis thaliana* was shown to be less viable in green peach aphids in infested leaves [6]. In chili plants, dietary effects were induced and volatile organic compounds were released with a repellent outcome versus infested *Bemisia tabaci* [7]. In *Brassica napus*, there was a decrease in survival rate and population growth parameters of immature *Plutella xylostella* due to *Brevicoryne brassicae* resistance [8].

The defense response in plants is induced by jasmonic acid (JA), salicylic acid (SA), and ethylene (ET) [9]. SA has been found to be involved in the defense against sucking-piercing insects, while JA has been found against chewing insects [10]. ET controls different processes associated with plant defense responses [11]. *Danaus plexippus* increases JA pathway activation but controls acquisition in SA in the case of the oleander aphids, *Aphis nerii*; JA caused the opposite impact in *Asclepias syriaca* [11]. Few previous studies have demonstrated the involvement of JA and SA in the induction of aphid response from enhanced expressions of genes like *PR-1*, *PR-2*, *CHIT1*, *LOX1*, and *PAL* that have been identified as responses induced by JA–SA, after aphid feeding [12,13].

Because of its feeding behavior, *Myzus persicae*, a major destructive pest of cucumber, maize, barley, wheat, and beans in China, has a direct impact on the yield and quality of the crops. Biotic and abiotic elicitors are the catalyst for plant defense response [14]. Different pathogens, including fungi, bacteria, viruses, and oomycetes, are associated with the elicitors. Proteins, glycoproteins, peptides, lipids, and oligosaccharides are the most common elicitors [15]. They comprise two main groups—race-specific groups that trigger a defense response only for host plants and those that lead to a general defense response for both host and non-host plants [16]. Due to the increased demand for food safety, quality elicitors have been studied as replacements for certain chemical pesticides [17,18,19,20].

PeBL1 is a broad spectrum, widely-specific elicitor studied in the A60 strain of *Brevibacillus laterosporus* and has been found to be able to activate resistance in plants through the JA and SA pathways. It triggers defense enzyme activation, strengthens cell walls, and increases the regulation of other defense-associated genes [21]. The pathogenicity of *B. laterosporus* is associated with and active against dipteran flies and mosquitoes, and it is related to a mixture of sporulated cultures with or without parasporal bodies [22]. A typical, morphologically marked spore surrounded by a strongly attached canoe-shaped parasporal body is the *B. laterosporus* anti-microbial species, and it is a pathogen of invertebrates. The potential for biocontrol in *B. laterosporus* includes not only phytopathogenic fungi and bacteria but also insects, nematodes, and mollusks [22]. The biocontrol potentials of insects in the orders of Coleoptera, Lepidoptera, and Diptera have been studied in entomopathogenic species [23]. Applications of the PeBL1 elicitor on cucumber seedlings were studied in the current study, as were the function and mechanism of the effects of PeBL1 on cucumber aphid control to assess the potential impact of PeBL1 on *M. persicae*. Trichomes were found in leaf the surface structure, and so the contents of JA and SA gene expression from JA and SA were carried out. Data on PeBL1’s function, mechanism, and effects in the control of cucumber aphid are herein provided.

## 2. Materials and Methods 

### 2.1. Aphid and Plant Preparation

*Myzus persicae* (Sulzer), commonly known as the green peach aphid, was collected from the cucumber field at the Chinese Academy of Agricultural Sciences in Beijing, China, and transferred to cucumber seedlings (*Cucumis sativus*). The aphid was reared in a chamber with 16:8 h light/dark photoperiod, 60% relative humidity (RH), and 23 ± 1 °C at the State Key Laboratory for Biology of Plant Diseases and Insect Pests, Institute of Plant Protection, Chinese Academy of Agricultural Sciences, No. 12 Zhong-Guan-Cun South Street, Beijing 100081, China. Cucumber (*C. sativus*) seeds were sterilized with 75% ethanol over 15–20 s and washed with distilled water, and then they were pre-soaked in distilled water 2–3 days before use.

### 2.2. Evaluation of PeBL1

PeBL1 was produced with the recombinant vector pET30-TEV/LIC in *Escherichia coli* BL21-DE3 (Novagen, Darmstadt, Germany). The pellets were removed, and the supernatant cells were resuspended and sonified by the ultrasonic disruptor. The supernatant was collected and filtered with filter paper (size 0.22 µm) after the solution was centrifuged at 12,000 rpm for 15 min. The Äkta Explorer Protein Purification System (Amersham Biosciences, Temecula, CA, USA), as described by Wang et al. [21], with a His-Trap HP column (GE Healthcare, Waukesha, WI, USA), used various loading buffers (A, B, C, and D) for the further purification of the elicitor protein PeBL1. Buffer A (50 Mm Tris-HCl, 8.0 pH), washed off other elicitors from the column quickly, and buffer B was used to stabilize the column (50 Mm Tris-HCl, 200 Mm NaCl). For the solution elution elicitor protein, Buffer C (50 Mm Tris-HCl, 200 Mm NaCl, and 20 Mm imidazole, pH 8.0), and elusion Buffer D (50 Mm Tris-HCl, 200 Mm NaCl, and 500 Mm imidazole, pH 8.0). Then the PeBL1 elicitor protein was desalted in a HiTrap desalting column (GE Healthcare, Waukesha, WI, USA), as described by Wang et al. [21]. The molecular mass of the purified elicitor protein was measured by a 12% SDS-PAGE resolving gel, and a GenStar M223 protein marker (~5–245 kDa) was used for the estimation of the molecular mass of the purified PeBL1 elicitor.

### 2.3. The Population of M. persicae 

Cucumber seeds and young seedlings were soaked for 24 h in four concentrations of the PeBL1 solution, i.e., 70.58, 42.34, 21.17, and 17.64 μg mL^−1^. Three seeds in a single pot were cultivated in organic soil (Flora Guard substrate). Three-week-old seedlings of cucumber with the different concentrations of the PeBL1 solution were sprayed after 7 days and then inoculated with 10–12 adults of *M. persicae* per plant after 24 h. For *M. persicae,* after inoculation, the number of settled aphids’ population, was recorded after every 5 days as described by Li et al. [24]. Water and 70.58 μg mL^−1^ of a buffer (50 mM Tris-HCl, pH 8.0) were tested for positive and negative controls. A CRD randomized statistical design was used. Transparent air-permeable cages were used to separate seedlings from each plant. The experiment was conducted twice with four replications.

### 2.4. The Intrinsic Rate of Increase of M. persicae

Cucumber seeds were soaked in 70.58 μg mL^−1^ of a purified protein solution for 24 h and then transferred for sprouting 2–3 days in distilled water in petri plates. A CRD randomized statistical design was used. Seedlings were sprayed after 24 h with 70.58 μg mL^−1^ of the PeBL1 purified protein solution. Inoculation with a freshly born *M. persicae* nymph was then carried out for every seedling. A glass tube cotton-gauze was used to separate all seedlings. Twice a day, the new-born aphid was observed to record the total time and number of offspring produced, which were removed every day. The same test was conducted on seeds and seedlings after 5 days. The experiment was repeated twice individually, with 30 replicates per treatment. The increase in each aphid’s intrinsic rate was measured by:

(1) rm = 0.738×(ln Md)/Td. 

*Md* is the number of nymph’s new-born in the development time equal to *T_d_*, which is the time between an aphid’s birth and its first reproduction

### 2.5. Feeding Preference of M. persicae Choice Test

Cucumber seeds and seedlings, as described in Section 2.2, were treated. The cucumber PeBL1-treated and control seedlings were put in a transparent breathable cage (60 × 60 × 60) cm with cross-touch leaves and a white cardboard bridge (12 × 4) cm connecting the base section of stems. A CRD randomized statistical design was used. Thirty wingless *M. persicae* adults in the center of the bridge were released. The experiment was repeated 15 times, and after 24 h, the aphids were counted on each seedling.

### 2.6. Detection of Aphid Feeding Activities by Electrical Penetration Graph (EPG)

As previously described, the cucumber seeds were soaked and germinated for 3–4 days in distilled water. Similarly, sized seedlings were then individually planted to organic soil until day 7. Twenty-four hours after the spraying of the seedlings, an electrical penetration graph (EPG; GIGA-8d) was used on wingless, 12–15-day-old, healthy adult *M. persicae*. Before the test, all aphids were starved for 1 h. The experiments were conducted daily for 4 h at the same time. An A B stylet was used for the determination and manual study of the aphid feeding waves. A wave identifier was previously described [25].

### 2.7. Aphid Bioassay

A bioassay of the PeBL1 elicitor was carried out with different concentrations of the protein purified solution, i.e., 70.58, 42.34, 21.17, and 17.64 μg mL^−1^, a positive control (water alone), and a negative control (70.58 μg mL^−1^ buffer) against *M. persicae* on the plants of cucumber. A Bradford assay was used to determine different protein concentrations. At the three-leaf stage of the cucumber plant, approximately 2–3 mL of PeBL1 was applied with a separate spray bottle until the solution drained off from plants. For positive and negative controls, waters and buffers (50 mM Tris-HCl, pH 8.0) were used. The plants were allowed to dry overnight, and 3–5 numbers of freshly molted 0–6 h aphids were allowed to feed on these plants. The time of nymph development was observed by consecutive observations at intervals of 3 h until the bioassays were completed for each instar as the total number of offspring produced by all aphid instars, while the number of days in which aphids lived was considered the longevity. Data were compared statistically by a factorial ANOVA and least significant difference (LSD) at α = 0.05. Bioassays were repeated independently at three non-identical temperature regimes (20, 24, 27 °C) by using 10 replicates per treatment.

### 2.8. Effect of PeBL1 on the Growth and Structure of Cucumber

Seven-day-old cucumber seedlings were treated in the same manner as above. One day after the seedlings were sprayed, the seeds were soaked for eight days. The central part for the first leaves was collected and tested, while 3.5% glutaraldehyde diluted into a 0.1 M phosphate buffer (pH 7.2) was used for sampling up to 48 h. For approximately 15 min, all samples were cleanly washed in a 0.1 M phosphate buffer (pH 7.2) and then submerged in 1 percent osmic acid for about 2 h five different times. A gradient of ethanol of 100, 95, 90, 80, 70, 60, 50, and 30% was used for 15 min. A Leica EM critical point dryer (CPD030; Leica Biosystems, Wetzlar, Germany) was used to dry all samples at critical points. A Hitachi H-7650 transmission electron microscope was used to monitor all samples, Total plant height (cm), total chlorophyll amount (SPAD), total fresh and dry weight, and the number of plant leaves, with 10 replicates per treatment, were measured in order to quantify the effect of PeBL1-treated settlements. A CRD randomized statistical design was used, and data were compared statistically by an ANOVA and LSD at α = 0.05.

### 2.9. HPLC/MS Detection of the Plant Hormone

As before, seeds and seven-day-old seedlings were treated. As mentioned above, approximately 0.5 g of the aerial part of the seedlings was collected to extract SA, JA, and ET [26]. A high-performing liquid chromatography spectrometer was used to inject some 20 μL of extraction (HPLC/MS; Shimazu Research Instruments, ODS-C18, 3 μm, 2.1 per 150 mm Kyoto, Japan). HPLC was performed at a flow rate of 0.2 mL min^−1^ with a mobile phase of 60% methanol, 40 °C column temperature, and 4 °C sample temperature. In the negative ion mode (SA m/z: 137.00; JA: 209.05) MS was set at the selected ion monitoring system (SIM) with a solvent temperature of 250 °C, a heat block temperature of 200 °C, a drying gas flow rate of 10 L min^−1^, a nebulizing gas flow of 1.5 L min^−1^, a detector voltage of 1.30 kV, and an interface voltage of −3.5 kV.

### 2.10. Expression of the Gene by Q-RT-PCR

TransGen Biotech (Beijing, China) kits were used for extracting RNA, synthesizing cDNA, and conducting a quantitative polymerase chain reaction in real-time (RT-qPCR) (ABI 7500 Real-Time PCR System). The excellence of RNA was calculated with an NP80 nano-photometer. The tested JA, SA, and ET genes were *ChIT1* (class III chitinases PR-3); *β-1*,*3-Glucanase* (beta-glucanase); *PAL1* (phenylalanine ammonia-lyase); *LOX1* (lipoxygenase multifunctional proteins), *PR1* (pathogenesis-related to protein 1), *cupi4* (cucumber pathogenesis-induced 4), and *PR2* (pathogenesis-related to protein 2); the ribosomal gene 18S was considered as the internal reference gene [27]. The primers of all pathways are listed in Table A1 of Appendix A. The application of the 2^−ΔΔCT^ was used to check the relative fold expression of the genes [28].

### 2.11. Analysis of Data 

Data from two treatments were compared statistically using an independent Leven’s test, and a two-tailed *t*-test and data obtained from three or more treatments were compared statistically by the LSD and an ANOVA. Statistix software version 8.1 (Analytical Software, Tallahassee, FL, USA) was used for statistical data analysis. Data on the fecundity of aphids were square-root transformed prior to analysis. In order to take out differences, a one-way factorial analysis of variance was performed among treatment factors such as the concentrations of PeBL1 elicitor and different temperature regimes, followed by the least significant difference test, at a probability of 95%. The expressions of genes (RT-qPCR) were obtained by the comparative CT (2^−ΔΔCT^) method. The Student’s *t*-test (*p* = 0.05) was used to compare fold changes in the plant samples treated with the elicitor and the buffer.

## 3. Results

### 3.1. Expression, Purification, and Evaluation of PeBL1 Elicitor Protein

**Figure 1:**
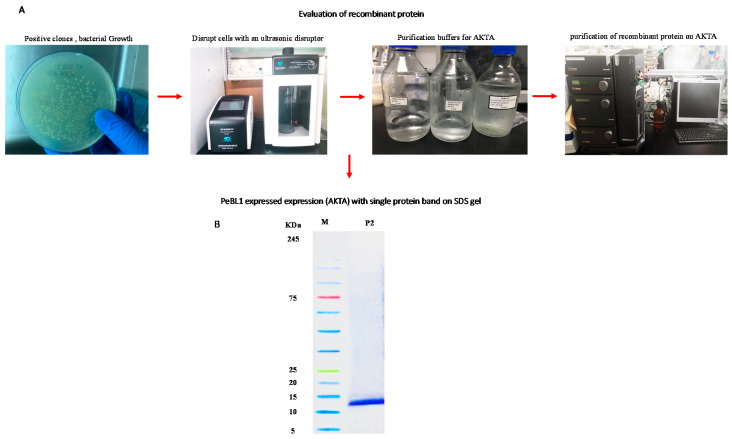
PeBL1 recombinant protein purification. (**A**) Evaluation of recombinant protein by using the Äkta explorer (**B**) His-Trap HP column used for the purification of total *E. coli* proteins. An elution buffer—25 mM Tris, 200 mM NaCl, 500 mM imidazole, and pH 8.0—was used to elute peak P2, which comprised recombinant PeBL1 with a flow rate of 5 mL/min. A HiTrap desalting column was used to load P2. The purified and desalted PeBL1 protein formed a single band of the molecular mass of 12 kDa on Tricine SDS-PAGE. Lane M: protein molecular mass marker; lane P2: peak P2.

The PET30-TEV/LIC recombinant vector was transformed into *E. coli* BL21 (DE3) cells. The expressed His6-PeBL1 was soluble in *E. coli* after successful transformation. PeBL1 was purified with the column of the His-Trap HP (GE Healthcare, Waukesha, WI) (Figure 1A) and was desalted, as previously described by Wang et al. [21], in the HiTrap column. A single band showed the characteristics of pure recombinant protein at 12 kDa on Tricine SDS-PAGE (Figure 1B).

### 3.2. Myzus persicae Performance Indoors 

**Figure 2:**
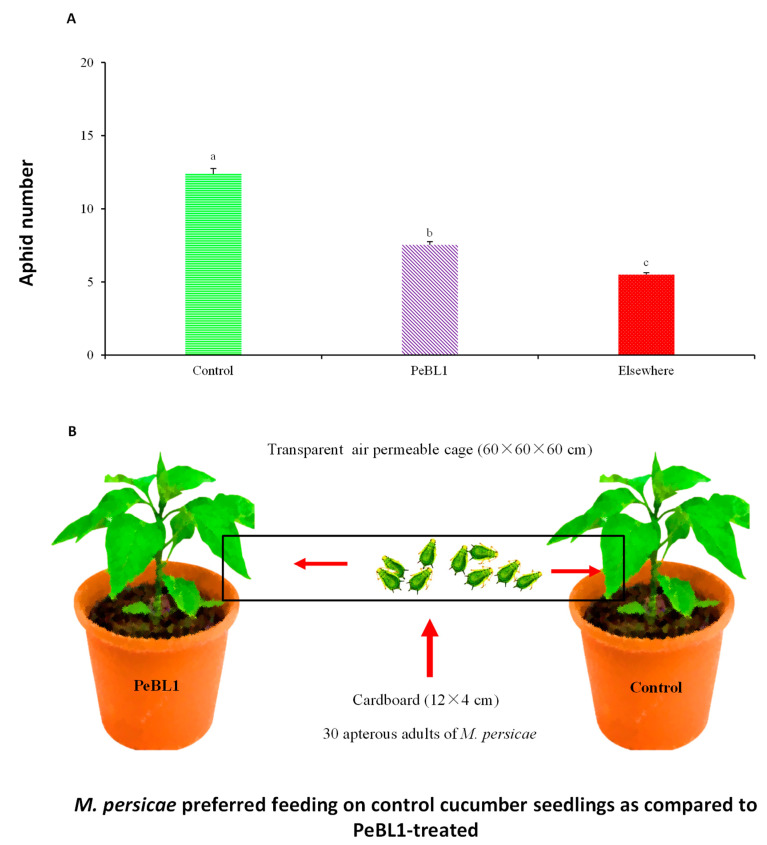
The number of *M. persicae* colonized on control and PeBL1-treated cucumber seedlings (**A**) 24 h after the infestation colonization of *M. persicae* (mean ± SD). Data were compared statistically by an ANOVA and LSD in Statistix, version 8.1. Significant differences can be seen in lower style alphabet letters among all treatments (*p = 0.05*) (**B**) *M. persicae* colonized and preferred feeding on control cucumber seedlings as compared to the PeBL1-treated seedlings.

PeBL1 induced resistance to cucumber aphid *M. persicae* in two separate ways. First, a settled aphid population decrease was observed in the PeBL1-treated cucumber seedlings (Table 1), and the percentage decreases in population count in PeBL1 treatment relative to the buffer and control treatments are indicated in Table A2, Table A3 and Table A4 of Appendix B. *M. persicae*, in the host selection assay, preferred to feed on the control cucumber seedlings. A day after the inoculation of the aphid and two days after spraying of the seedlings, the amount of *M. persicae* colonizing PeBL1-treated plants was significantly lower than the control and “Elsewhere”—the latter of which is aphid colonization in places other than buffer-and PeBL1-treated areas (Table A5 of Appendix C). Based on their feeding behavior, some aphids showed colonization in areas opposite to that treated with buffer and PeBL1, (Figure 2A,B). Second, in the case of PeBL1 treatment, the developmental time of the cucumber aphid was extended more compared to the control, whereas the everyday reproductive abilities of *M. persicae* that were fed on seedlings treated with PeBL1 were reduced (second and third nymphal instars). Second and third generations experienced lower growth rates (Table 2).

### 3.3. M. persicae Feeding Behavior by EPG

An EPG provided a general illustration of the cucumber resistance factors. The feeding behavior of *M. persicae* in seedlings treated with PeBL1 was significantly altered. EPG data (Table 3) showed that the probing period, the length of C (pathway operation in all tissues), and the sum of *M. persicae* Pd (potential decrease in cell punctures) were significantly reduced, whereas the period of non-probe time before the first E (phloem-feeding activity) and the total duration of F (penetration problems) in the PeBL1-treated cucumber seedlings increased considerably. No electrical contact between the aphid stylet and the plant was indicated during the non-probing time [29]. In the PeBL1 treatment, the non-probing time before the first E was dramatically increased, suggesting a repellent or deterrent surface factor in the PeBL1-treated cucumber seedlings. C waves represent style motion in intercellular space and may constitute a plant mechanical barrier [30]. The shorter C waves (<3 min) that were detected, the greater the mechanical difficulty in seedlings treated with PeBL1. Additionally, a lower Pd number (cell puncture) was associated with the plant’s aphid resistance, which could have been due to mechanical difficulty (in this study, in the PeBL1-treated cucumber seedlings). The wave E1 (saliva injection during phloem-feeding activities) indicated saliva injection by aphids into sieve elements. In contrast, the E2 wave (sap-sucking during phloem-feeding activities) showed phloem sap injection with concurrent salivation, which could have reflected a mesophyll or vascular resistance factor [31]. In the sieve element [24,31] the extended E1 indicated more plugging or defense compounds. There was, however, no substantial difference between the control and PeBL1 treatments in the E2 period, indicating no or low variability in phloem compounds to confer resistance to *M. persicae*. However, the period of the F wave in the PeBL1-treated cucumber seedlings was higher, indicating that PeBL1 induced an enhanced mechanical defense. The EPG results suggested that the resistance induced by PeBL1 was mainly due to the modification of the physical defense.

### 3.4. Impact of PeBL1 Elicitor on Aphid’s Nymphal Development Time

**Figure 3:**
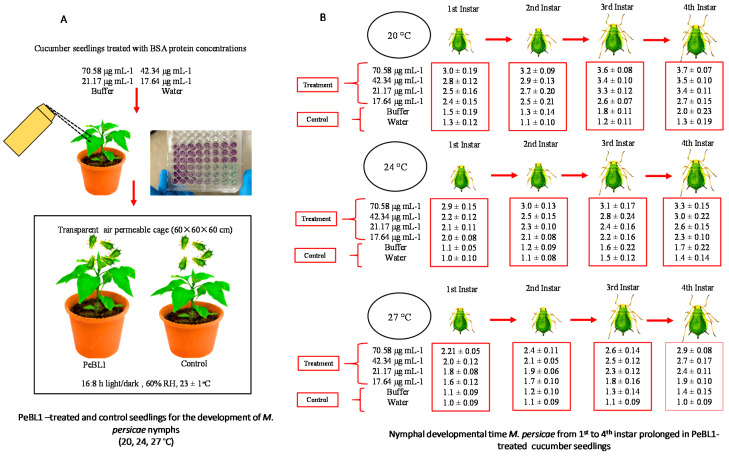
Prolonged nymphal development time of aphid instars (1st, 2nd, 3rd, and 4th). (**A**) Cucumber seedlings treated with control, buffer, and four PeBL1 protein concentrations prepared by BSA. (**B**) Nymphal developmental time of *M. persicae* from the prolonged 1st to 4th instar’s in PeBL1-treated cucumber seedlings. Data are shown as the mean (±SE) of different nymphal instars of (*M. persicae*) on cucumber plants by the PeBL1 elicitor protein at different concentrations and different temperature regimes (*n* = 10). Data were compared statistically by a factorial ANOVA and LSD at α = 0.05 in Statistix, version 8.1. Different alphabets above bar tops specify significant differences among treatments.

Factorial analysis showed an impact on the overall developmental time of *M. persicae* on various PeBL1 concentrations in three different temperature regimes and their interaction. A differential trend was identified on the developmental time of nymphs for the elicitor effect at a different temperature, as shown in Table A6, Table A7, Table A8, Table A9 and Table A10 of Appendix D. With increasing concentrations of PeBL1, (Figure 3A) the development time of each nymphal instar was extended. For the fourth nymphal instar at a high concentration (70.58 μg mL^−1^), the maximum development time was 3.7 d at the low temperature of 20 °C. At the low concertation (17.64 μg mL^−1^) for the first instar at the high temperature of 27 °C, the minimum nymph growth time was recorded. The time of nymph development (Figure 3B) differed in the buffer control and water-treated control. In general, the nymphal development time of all instars at the low temperature was higher than at medium or high temperatures. At each concentration and temperature of the elicitor, the maximum elongation of time was observed at fourth instar. However, for the first, second, third, and fourth instar aphid concentration of the elicitor, PeBL1 showed significance. Likewise, the effect of temperature regimes also had a significant impact on the developmental time of nymph of the first, second, third, and fourth instar aphids. Afterwards, nymphal aphid development time showed little difference across their shared interface. After all, the nymphal development time did not show any fluctuation with their mutual interaction.

### 3.5. Effect of PeBL1 Elicitor on the Aphid’s Fecundity

**Figure 4:**
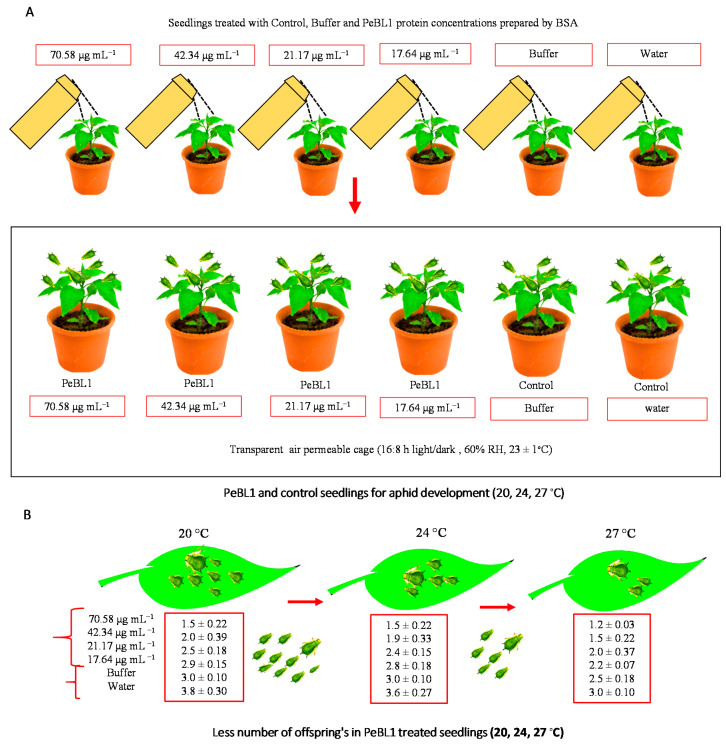
*M. persicae* fecundity reduced in PeBL1-treated cucumber seedlings. (**A**) Seedlings treated with control, buffer, and PeBL1 protein concentrations prepared by BSA. (**B**) PeBL1 and control seedlings for aphid development (20, 24, and 27 °C). Average fecundity of *M. persicae* on cucumber plant in relation to various PeBL1 concentrations at different temperature regimes (*n* = 10). Data are shown as mean (±SE). Data were compared statistically by a factorial ANOVA and LSD at α = 0.05 in Statistix, version 8.1. Letters on each bar’s top show significant differences among treatments.

**Figure 5:**
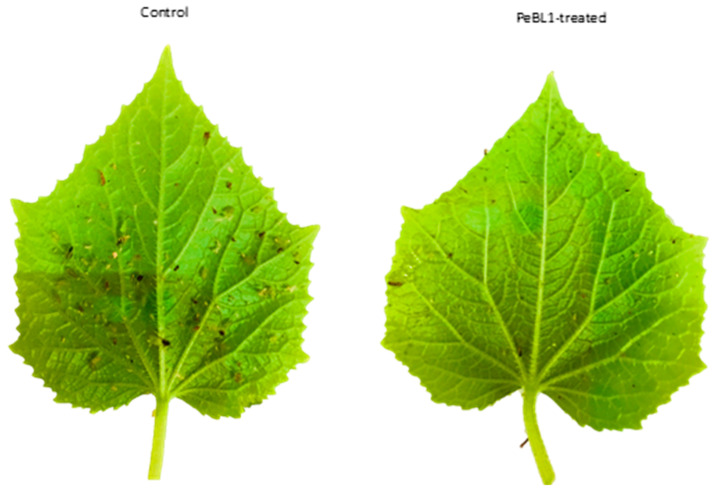
*Myzus persicae* that fed on PeBL1 showed a reduction in the number of offspring as compared to the control plants.

The figures show that the concentrations of PeBL1 (F_5.162_ = 83.82; *p* < 0.0001) and temperature regimes (F_2.162_ = 8.97; *p* < 0.0002) were significantly affected by aphid fecundity, as shown in Appendix E of Table A11. (Figure 4A,B) shows that *M. persicae* produced less offspring than those that feed on (water) positive and (buffer) negatively treated cucumber plants in contrast to the PeBL1-treated seedlings (Figure 5). However, a minimum fecundity at a maximum temperature was observed at 27 °C, and a maximum fecundity at a minimum temperature of 20 °C was recorded.

### 3.6. Effect of PeBL1 on the Growth and Structure of Cucumber

**Figure 6:**
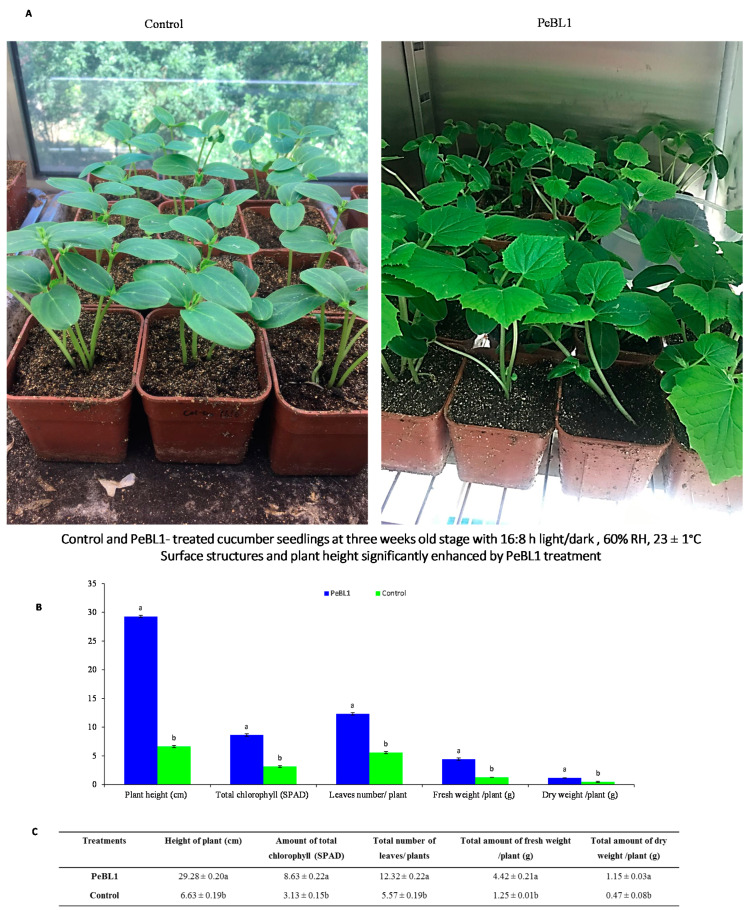
Influence of PeBL1 on the growth of PeBL1-treated and control cucumber seedlings. (**A**) Plant height increased in PeBL1-treated cucumber seedlings as compared to control (**B**) Physical characteristics of PeBL1-treated seedlings enhanced as compared to control (**C**) Data were shown in mean (±SD) of cucumber plants in PeBL1-treated and control seedlings (n = 10). Data were compared statistically by one-way analysis of variance (ANOVA) and Least significant difference (LSD) in Statistix 8.1 version. Different lower style alphabets letters indicate significant differences among treatments (*p* = 0.05).

The plant height and surface structure of the cucumber leaves were greatly modified by PeBL1 (Figure 6). A similar trend was also found in the SPAD, the total number of leaves, and plant fresh and dry weight (Table A12, Table A13, Table A14, Table A15 and Table A16 of Appendix F). In PeBL1-treated seedlings, plant height was greater than that of the control. The surface of cucumber leaves was significantly modified by PeBL1 elicitor protein, seedlings treated with PeBL1 exhibited more trichomes as compared to control in PeBL1 treated 60.84 ± 0.52 mm^−2^, while in control treatment it was 34.30 ± 0.10 mm^−2^. A more sophisticated wax structure was formed that gave rise to a significantly better surface environment, one that trait is considered to be unfavorable to aphid settlement [32].

### 3.7. SA, JA and ET Accumulations in PeBL1-Treated Cucumber Seedlings

**Figure 7:**
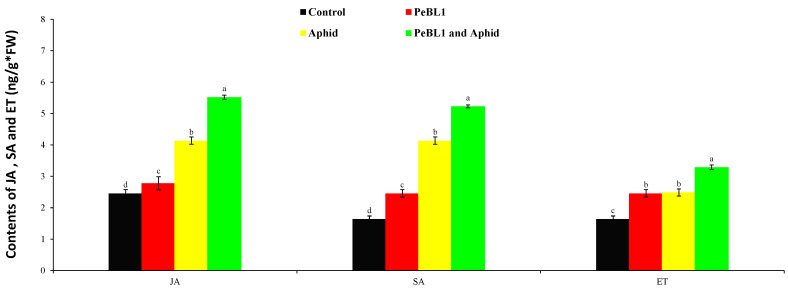
Jasmonic acid (JA), salicylic acid (SA), and ethylene (ET) contents in cucumber seedlings (mean ± SD). Treatment with PeBL1 was collected one day after spraying. In both treatments, the aphids were inoculated one day after seedlings were sprayed, and the samples were collected one day after inoculation. Data were compared statistically by the LSD, ANOVA, and Leven’s test using Statistix, version 8.1. Different lower-case letters show significant differences among various treatments performed in JA, SA, or ET (*p* = 0.05).

To analyze the relation of JA, SA, and ET with the cuticular wax deposition and increase in the trichome density of PeBL1, the aphid infestation or both were analyzed. PeBL1 showed a very high accumulation of JA, SA, and ET in seedlings (Figure 7). All three pathways of signaling were found to participate in aphid-induced cucumber resistance [33]. In addition, JA, SA, and ET accumulated in PeBL1-treated plants, suggesting that the protein elicitor at least partially induced the defense response in cucumber plants. Numbers, infestation rates, and aphid species are known to affect JA, SA, and ET inductions [34,35].

### 3.8. Relative Fold Expressions of Defense-Related Genes

**Figure 8:**
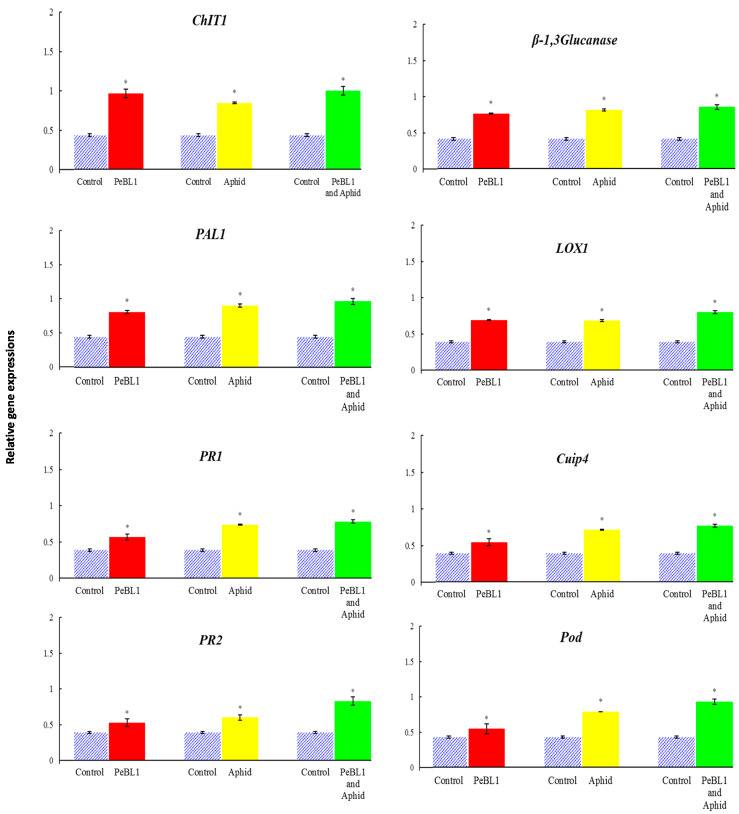
Relative expression of plant defense from the JA, SA, and ET pathway genes found after treatment with the PeBL1 elicitor, PeBL1 and aphid, and aphid infestation alone. For each gene, an asterisk on the bar shows a significant difference from the buffer control, as found by Student’s *t*-test (*p* < 0.05).

PeBL1 enhanced the defense mechanism in cucumber seedlings. *ChIT1*, *β-1*,*3-Glucanase*, *PAL1*, *LOX1, PR1, cupi4*, *PR2,* and *Pod* were selected as test genes for defense pathways. All the genes were up-regulated by treatment with PeBL1 and aphid infestation (Figure 8); the transcripts of all genes were found to be statistically greater with the PeBL1 treatment than with the other two treatments. The idea was that PeBL1 treatment enhanced induced resistance to aphid infestation. The improved transcript of test genes of JA and SA also demonstrated their roles for the JA and SA pathways in aphid resistance in wheat, which was proven by Moron and Thompson. The highest expressed gene was *ChIT1*, followed by *β-1*, *3-Glucanase*, *PR1*, *LOX1*, *PAL1*, *cupi4*, *PR2*, and *Pod*. The Log_2_ of all JA, SA, and ET test genes was computed with fold change expression values (Table 4) showed that aphid resistance was due to the transcription of the test genes.

## 4. Discussion

The use of elicitors is a new biological tool for the management of insect pests, as they play a dynamic role in the defense and signaling mechanisms of plants under the attack of sap-feeding insects [17,19,20], Numerous *B. laterosporus* strain have demonstrated various broad-spectrum anti-microbial activities, acting as anti-microbial peptides in microbes such as bacteria and fungi. They can enter into the cell and transfer to the cytoplasm and nucleus to interrupt protein synthesis by mixing up DNA and RNA [36,37]. A significant source of elicitors such as PAMPs or MAMPs [38] is pathogenic bacteria and fungi, whether necrotrophic or biotrophic. The potential activities of PeBL1 derived from *B. laterosporus* strain A60 were demonstrated in this study for *M. persicae* management. Certain studies have previously shown chemical elicitors significantly reduced the activity of herbivorous pests in cucumber crops by applying chemical elicitors, such as methyl-jasmonate, benzothiadiazole, and other plant defenses, including proteinase inhibitors [39]. Results from this study confirmed previous results that the use of methyl salicylate elicitor reduced the soybean aphid *Aphis glycines* by up to 40% [38,39]. Here, bioassays showed that population development on PeBL1-treated cucumber plants was significantly slower compared to the buffer and control. Previous studies have shown a negative influence of exogenous applications of elicitors, including MJ, JA, and BTH, on the population growth and fitness of different aphid species, an effect confirmed by the present findings [39]. Similarly, a biocontrol potential was discovered against various Diptera, Coleoptera, and Lepidoptera, as well as against nematodes and mollusks [23,40]. The current study showed the ability of PeBL1 to suppress herbivores by influencing population and growth parameters. Trichomes are the first lines of physical resistance to pathogenic microorganisms and herbivores. These hairy adjuncts of plant epidermal cells affect the herbivores’ morphology and the density role of trichomes in *Solanum* spp., i.e., seven trichomes with two major defense-related effects, have been tested [40]. First, a plant surface represents a physical barrier because its thick matte hair provides energy, limits feeding capacity, and reduces access to the surface by insects. Excessively hairy plants, such as *Solanum hirsutum*, are avoided by *M. persicae*. Trichomes are also associated with the basic defensive mechanism of the tomato plant, as the surface area covered by the epidermal cell appendages of unicellular or multicellular hairs provide resistance to a variety of pests due to the “pubescence” of a plant. Leaf beetle (Coleoptera: Chrysomelidae) settlement with thick trichomes was reduced in soybeans compared with the trichome-removed plants, which attracted more beetles [41,42,43]. PeBL1 reduced disease severity, triggering a photosynthesis process that mirrored the characteristics of growth of plants [44], and it enhanced induced-resistance in the PeBL1-treated cucumber seedlings as well.

Compared to the controls, the PeBL1-treated seedlings and leaves had more trichomes. The PeBL1-treated cucumber seedlings and leaflets were reported to have inhibited the reproduction and settlement of aphids with an increased number of trichomes. The feeding activity of *Leptinotarsa decemlineata* was negatively affected by a high density of trichomes. Another key part of physical barrier lignin is the cell wall, which underpins plant resistance and is an indicator for the improvement of the cell wall [45]. Aphid tolerance in chrysanthemum was amended by an enhanced lignin content [46]. The physical defenses of plants include, in response to biotic and abiotic stress, trichomes and wax production. Their establishment can be induced by direct damage, e.g., as induced by leaf-cuts, methoxyfenozide, and manganese [47,48]. The use of exogenous phytohormones, MJ, or JA can also affect cuticular wax deposition and trichome density, as shown in *Arabidopsis* and tomatoes, respectively [49]. The SA application was due to wax deposition in *Brassica napus* [50]. Accumulations of SA and JA in PeBL1-treated cucumber plants can, therefore, be hypothesized as being related to increased trichome density and the deposition of cuticular wax. In addition, the treatment of the PeBL1 elicitor had adverse effects on aphid fecundity. PeBL1-treated plants produced significantly fewer aphids than the buffered and controlled seedlings. The results were consistent with previous studies showing that exogenous SA and MJ have caused lower mean lifetime fecundity in aphids [38]. Therefore, optimum temperatures (e.g., 24 °C) demonstrated a maximum aphid fecundity, with the minimum fecundity at higher temperatures (27 °C) due to a decreased metabolic rate [51]. Similarly, a variance analysis showed that in PeBL1-treated plants, the development time of nymphs was extended compared to the control; even at a lower temperature (20 °C), the maximum nymphal development time was observed, indicating that a one-degree temperature increase affected the life cycle of the insect [52]. Additional studies need to be conducted to understand the underlying mechanism of PeBL1 in cucumbers, in particular its effect on fecundity and nymphal development time.

Additionally, JA, SA, and ET increased marker gene transcriptions, signaling that they play an essential role in cucumber aphid resistance. After aphid infestation in *Arabidopsis*, the transcript genes *CHIT1*, *β-1*,*3-glucanase*, *PR1*, *LOX1*, *PAL1*, *cupi4*, *PR2*, and *Pod* were significantly increased. Actin is a structural component in the plant cell wall that is depolymerized via the regulation of cell and cross-linking [53,54]. Actin depolymerization is negatively related to aphid fecundity and population [55]. JA, SA, and ET molecules impart resistance to insect herbivorous diseases and pathogens, which enhances plant defense responses [12,56,57,58]. All JA, SA, and ET test genes showed significant and robust regulation [12]. *PAL1* coding for ammonia-lyase phenylalanine is involved in cell wall construction, as demonstrated in *Arabidopsis* [59]. JA and *LOX1* up-regulation occur in cucumber plant *Pseudomonas* injection [59,60]. *PR1* and *PR2* are systemically acquired (SAR) pathogenesis-related proteins [13,61,62]. *Cupi4* genes with antibacterial characteristics also lead to the induction of hypersensitive reactions in the infected tissues of plants [63,64]. The presence of *CHIT1* (PR-3 family) genes indicates fungal cell wall damage in plants due to antifungal activity [13,65]. *β-1*, *3-Glucanase* (beta-glucanase) codes show a plant-friendly defense mechanism against several microorganisms, as well as an increase in *β-1*,*3-glucanase* in *P. melonis* fungal cell walls, which leads to the inhibition of disease growth in infected plants [66,67]. *Pod* codes peroxidase, which indicates that the acclimation of the *Pod* will mediate aphid resistance in cucumbers [68]. Experimental findings from this research confirm that the activation of *Pod* by *M. persicae* induces genes associated with the defense pathway [69].

## 5. Conclusions

Herein, we present data on aphid resistance in cucumbers with the prolonged developmental time of the first to fourth nymphal instars, related to a lower fecundity of *M. persicae*. Increased PeBL1 concentrations were found to affect aphid colonization. The resistance factors were verified by the increased number of trichomes and wax amounts, which were mainly involved in mechanical defenses. Likewise, an EPG study confirmed that the resistance induced by PeBL1 was mainly due to the modification of the physical defense and increased number of trichomes, and wax composition affected aphid feeding behavior in PeBL1-treated. Moreover, our study focused on the effect of PeBL1 on the growth and structure of cucumber, and we found that increased plant height and modified surface structures of the cucumber leaves were greatly influenced by PeBL1. We also confirmed the role of PeBL1 in physical defense against *M. persicae*. The physical defense response induced by PeBL1, JA, SA, and ET contributed to a comprehensive plant physical response.

However, some issues need to be resolved in the future, e.g., “how JA, SA and ET work resistance induction,” and “whether or not other plant hormones are involved.” Nevertheless, the current study showed that PeBL1 isolated from *B. laterosporus* A60 strain could be applied to cucumber seed and seedlings to protect plants against *M. persicae*.

## Figures and Tables

**Table 1:** *M. persicae* settled population data in PeBL1-treated, control, and buffer-treated cucumber seedlings showed differences of *M. persicae* number after every 5, 10, and 15 days.

Days after Aphid Inoculation	Control	Buffer	PeBL1
5	51.99 ± 0.55 b	60.941 ± 0.28 a	44.70 ± 1.19 c
10	106.39 ± 1.22 b	122.18 ± 0.91 a	87.763 ± 1.98 c
15	216.70 ± 0.97 b	250.35 ± 1.07 a	173.80 ± 2.01 c

Note: Data are shown as mean ± SD. Data were compared statistically by an ANOVA and LSD in Statistix software, version 8.1. Significant differences can be seen in letters in rows at all treated samples after the inoculation of the aphid on the same day (*p* = 0.05).

**Table 2:** Developmental time, reproduction capacity, and increase of the intrinsic rate of *M. persicae* in PeBL1-treated and control seedlings of cucumber.

Generations of *M. persicae*	Td (Day)		No of Nymphs per Day		r_m_	
	Control	PeBL1	Control	PeBL1	Control	PeBL1
1st	6.82 ± 0.31	6.48 ± 0.19	3.51 ± 0.06	3.21 ± 0.09	0.41 ± 0.01	0.37 ± 0.02
2nd	6.13 ± 0.28	6.24 ± 0.22	2.68 ± 0.10	2.24 ± 0.12	0.33 ± 0.01	0.26 ± 0.01 *
3rd	6.92 ± 0.35	6.64 ± 0.30	1.76 ± 0.13	1.44 ± 0.06	0.18 ± 0.00	0.16 ± 0.01 *

Note: Data are shown as mean ± SD. Td represents development time, Number of nymphs per day represents average reproduction ability, and r_m_ is the increase of intrinsic rate. Data were compared statistically with an ANOVA and LSD in Statistix, version 8.1. Asterisks show difference between PeBL1 and control treatments *; (*p* = 0.05).

**Table 3:** Electrical penetration graph (EPG) data of *M. persicae* on PeBL1-treated and control cucumber seedlings.

EPG Parameters	Control *(N = 20)*	PeBL1 *(N = 20)*
Total probing time (h)	3.43 ± 0.03	2.50 ± 0.04 *
Number of C	14.43 ± 0.06	23.37 ± 0.45
Number of short probes (C < 3 min)	6.34 ± 0.41	21.19 ± 0.45
Duration of non-probe period before the 1st E (h)	2.45 ± 0.05	2.49 ± 0.05 *
Number of pd	73.09 ± 0.43	36.53 ± 0.20
Mean duration of Pd (s)	5.71 ± 0.07	5.60 ± 0.08
Number of E1	3.44 ± 0.06	2.75 ± 0.16
Mean duration of E1 (min)	7.12 ± 0.29	10.83 ± 0.23
Number of E2	0.88 ± 0.10	0.69 ± 0.13
Mean duration of E2 (h)	28.77 ± 0.23	41.91 ± 0.20
Number of G	0.90 ± 0.17	0.84 ± 0.10
Mean Duration of G (min)	18.19 ± 0.28	14.32 ± 0.27
Number of F	3.52 ± 0.25	1.77 ± 0.19
mean duration of F (min)	22.48 ± 0.38	50.89 ± 0.23

Note: Data are shown as mean ± SD. C stands for pathway activities, Pd stands for potential drop, E stands for phloem-feeding activities, F stands for penetration problem, G stands for xylem feeding activities, E1 stands for saliva injection, and E2 stands for sap-sucking. Data were compared statistically by an independent *t*-test with two tails and Levene’s test in Statistix, version 8.1. Asterisks indicate significant differences between PeBL1 treatment and control with the same parameters of * (*p* = 0.05).

**Table 4:** Log_2_ of fold change expressions all test genes involved in the JA, SA, and ET pathways after the PeBL1 elicitor application, PeBL1 and aphid, and aphid infestation alone.

Log_2_Fc (Treated/Untreated)	PeBL1	Aphid	PeBL1 and Aphid
*ChIT1*	1.15	0.96	1.20
*β-1*,*3Glucanase*	0.89	0.99	1.05
*PAL1*	0.85	1.01	1.10
*LOX1*	0.83	0.81	1.04
*PR1*	0.55	0.93	1.01
*Cupi4*	0.47	0.87	0.97
*PR2*	0.43	0.62	1.09
*Pod*	0.35	0.89	1.13

## Appendix A

**Table A1:** Primers of all test genes JA, SA, and ET involved in plant defense.

Test Gene	Forward Sequence (5′...... 3′)	Reverse Sequence (5′...... 3′)
*CHIT1*	TGGTCACTGCAACCCTGACA	AGGGTGAAAGCAAGAAGAGC
*β-1*,*3Glucanase*	TCAATTATCAAAACTTGTTCGATGC	AACCCTCTGGTAGGACAACAAC
*PAL1*	ATGGAGGCAACTTCCAAGGA	CCATGGCAATCTCAGCACCT
*PR1*	TGCTCAACAA ATGCGAACC	TCATCCACCCACAACTGAAC
*LOX1*	CTCTTGGGTGGTGGTGTTTC	TGGTGGGATTGAAGTTAGCC
*Cupi4*	TCACTGTGGTGTGTGCTCTC	ACTCAA GCCATTGCCTTCCA
*PR2*	TCAAGGAAGGTTCAGGGATG	TCGGTGATCCATTCTTCACA
*pod*	TCAGGATGGGAAATCTCGAC	CGTGGCCAACTCATACACAC
*18S*	GTGACGGGTGACGGAGAATT	GACACTAATGCGCCCGGTAT

## Appendix B

**Table A2:** Differences of *M. persicae* number after 5 days in PeBL1-treated, buffer, and control cucumber seedlings.

SOV	DF	SS	MS	*F*-Value	*p*-Value
Treatments	2	1323.43	661.713	110	0.0001
Error	27	162.24	6.009		
Total	29	1485.67			
Grand Mean/CV	2.45/28.49				

**Table A3:** Differences of *M. persicae* number after 10 days in PeBL1-treated, buffer, and control cucumber seedlings.

SOV	DF	SS	MS	*F*-Value	*p*-Value
Treatments	2	5938.56	2969.28	143	0.0001
Error	27	560.46	20.76		
Total	29	6499.02			
Grand Mean/CV	105.45/4.32				

**Table A4:** Differences of *M. persicae* number after 15 days in PeBL1-treated, buffer, and control cucumber seedlings.

SOV	DF	SS	MS	*F*-Value	*p*-Value
Treatments	2	29442.1	14721.1	722	0.0002
Error	27	550.3	20.4		
Total	29	29992.4			
Grand Mean/CV	213.62/2.11				

## Appendix C

**Table A5:** Number of *M. persicae* colonized on control cucumber seedlings, PeBL1-treated cucumber seedlings, and elsewhere.

SOV	DF	SS	MS	*F*-Value	*p*-Value
Treatments	2	498.05	249.02	561	0.0001
Error	27	11.97	0.44		
Total	29	510.02			
Grand Mean/CV	8.83/7.54				

## Appendix D

**Table A6:** Nymphal development time of 1st instar (*M. persicae*) in relation to PeBL1 elicitor and temperature.

SOV	DF	SS	MS	*F*-Value	*p*-Value
Conc.	5	53.22	10.64	72.46	0.0001
Temp.	2	10.93	5.46	37.23	0.0001
Conc. × Temp.	10	3.32	0.33	2.26	0.0167
Error	162	23.79	0.14		
Total	179	91.28			
Grand Mean/CV	1.99/19.32				

**Table A7:** Nymphal development time of 2nd instar (*M. persicae*) in relation to PeBL1 elicitor and temperature.

SOV	DF	SS	MS	*F*-Value	*p*-Value
Conc.	5	67.25	13.45	94.87	0.0000
Temp.	2	10.53	5.26	37.16	0.0000
Conc. × Temp.	10	3.75	0.37	2.65	0.0000
Error	162	22.96	0.14		
Total	179	104.50			
Grand Mean/CV	2.05/18.33				

**Table A8:** Nymphal development time of 3rd instar (*M. persicae*) in relation to PeBL1 elicitor and temperature.

SOV	DF	SS	MS	*F*-Value	*p*-Value
Conc.	5	75.06	15.01	75.63	0.0001
Temp.	2	19.93	9.96	50.21	0.0001
Conc. × Temp.	10	2.33	0.23	1.18	0.3103
Error	162	32.15	0.19		
Total	179	129.49			
Grand Mean/CV	2.05/18.33				

**Table A9:** Nymphal development time of 4th instar (*M. persicae*) in relation to PeBL1 elicitor and temperature.

SOV	DF	SS	MS	*F*-Value	*p*-Value
Conc.	5	83.88	16.57	74.27	0.0001
Temp.	2	18.28	9.14	40.97	0.0001
Conc. × Temp.	10	1.33	0.13	0.60	0.8131
Error	162	36.15	0.22		
Total	179	138.62			
Grand Mean/CV	2.45/28.49				

**Table A10:** Nymphal development time of overall (*M. persicae*) in relation to PeBL1 elicitor and temperature.

SOV	DF	SS	MS	*F*-Value	*p*-Value
Conc.	5	1096.89	219.37	199.77	0.0001
Temp.	2	231.52	115.76	105.41	0.0001
Conc. × Temp.	10	30.25	3.052	2.78	0.0034
Error	162	177.90	2.73		
Total	179	1536.84			
Grand Mean/CV	8.89/11.79				

## Appendix E

**Table A11:** Fecundity of *M. persicae* in relation to PeBL1 elicitor and temperature (ANOVA).

SOV	DF	SS	MS	*F*-Value	*p*-Value
Conc.	5	83.82	16.7644	34.14	0.0000
Temp.	2	8.973	4.4864	9.14	0.0002
Conc. × Temp.	10	1.952	0.1952	0.40	0.9463
Error	162	79.548	0.4910		
Total	179	174.294			
Grand Mean/CV	2.45/28.49				

## Appendix F

**Table A12:** Influence on the plant height of PeBL1-treated and control cucumber seedlings.

SOV	DF	SS	MS	*F*-Value	*p*-Value
Treatments	1	2567.60	2567.60	67921	0.0001
Error	18	0.68	0.04		
Total	19	2568.29			
Grand Mean/CV	17.95/1.08				

**Table A13:** Influence on the total amount of chlorophyll (SPAD) of PeBL1-treated and control cucumber seedlings.

SOV	DF	SS	MS	*F*-Value	*p*-Value
Treatments	1	136.86	136.869	3681	0.0002
Error	18	0.669	0.037		
Total	19	137.538			
Grand Mean/CV	6.04/3.19				

**Table A14:** Influence on the total number of leaves/plants of PeBL1-treated and control cucumber seedlings.

SOV	DF	SS	MS	*F*-Value	*p*-Value
Treatments	1	227.94	227.94	5402	0.0002
Error	18	0.760	0.042		
Total	19	228.70			
Grand Mean/CV	8.94/2.30				

**Table A15:** Influence on the total amount of fresh weight per plant (g) of PeBL1-treated, and control cucumber seedlings.

SOV	DF	SS	MS	*F*-Value	*p*-Value
Treatments	1	50.27	50.27	2377	0.0001
Error	18	0.38	0.02		
Total	19	50.65			
Grand Mean/CV	2.83/5.13				

**Table A16:** Influence on the total amount of dry weight per plant (g) of PeBL1-treated, and control cucumber seedlings.

SOV	DF	SS	MS	*F*-Value	*p*-Value
Treatments	1	2.32	2.32	698	0.0564
Error	18	0.05	0.0033		
Total	19	2.38			
Grand Mean/CV	0.81/7.13				
